# Spasm of the Glottis—Convulsive Croup

**Published:** 1831-04-01

**Authors:** 


### SPASM OF THE GLOTTIS—CONVULSIVE CROUP.

I. OBSERVATIONS ON A PECULIAR CONVULSIVE DISEASE AFFECTING YOUNG CHILDREN, WHICH MAY BE TERMED "SPASM OF THE GLOTTIS." By *H. Marsh*, M.D. (Dublin Hosp. Rept.)

II. CASES OF THE "PECULIAR SPECIES OF CONVULSION," DESCRIBED BY THE LATE DR. J. CLARKE, &c. By *Marshall Hall*, M.D. (Med. and Phys. Journ.)

III. CASE OF CARPO-PEDAL SPASM. (Med.-Chir. Journ.)

SINCE Dr. Clarke's observations on this peculiar disease, a great many detached cases, remarks, and conjectures have been published by various writers, especially Dr. Cheyne, Dr. Kellie, Mr. Porter, Mr. Pretty, &c.—to which list we may now add Drs. Marsh and Hall. The subject is interesting, and is still open to further investigation.

The disease in question appears to consist primarily in spasm, occurring suddenly, and affecting the muscles of the glottis. As it increases in severity, other muscles, particularly those of the fingers and toes, become progressively engaged—and ultimately, if neglected or mistreated leads to severe and general convulsions. This complaint is often overlooked or mistaken; while the principles of treatment, not always correct, have received but little attention. Dr. Marsh, whose papers we shall first notice, has selected a few cases from his notes, the leading facts of which only are stated. They, however, are sufficient to elucidate the characteristic features of the disease, and the principles of treatment. He remarks that it will be prudent to contemplate the disease in its mildest form, first—and afterwards to view it in its severer aspects, and in its more complicated characters.

"Indeed, in the study of spasmodic affections, it is important to learn, first, their essential characters, afterwards the functional derangements or diseases with which they are observed most frequently to co-exist; thus, for instance, in the treatment of Chorea, if complicated with derangement of the digestive organs, the purgative treatment recommended by Dr. Hamilton will often be effectual; but, if there be no such complication, as observation often proves to be the fact, this mode of treatment becomes worse than useless; it really adds to the disease which it is intended to relieve, and augments the convulsive movements of the muscles; hence it happens that many cases of Chorea which have resisted empirical courses of purgative medicines, have yielded speedily to local applications over the origin of the nerves which supply the muscles affected, combined with a decidedly tonic treatment. The spasmodic affection, now under consideration, requires to be studied in the same manner. It stands sometimes alone, no discoverable source of irritation, no other perceptible disease co-existing with it: more frequently, however, it is complicated with painful dentition, with derangement of the digestive functions, with a cachectic state of the system induced by an impure atmosphere, with fever, and occasionally with effusion into the ventricles of the brain; the consideration of these various complications bears most importantly upon the treatment." 602.

*Case* 1. This was a child eleven months old, very healthy, and well thriven, who had been for some time affected in the following manner.

"It had been observed, now and again, to awake suddenly from sleep in a state of alarm and agitation, to struggle for breath, and, after repeated efforts, to recover from the paroxysm, with a long and sonorous inspiration; the convulsive effort was described to be severe, and the face to become swollen and purplish: these attacks occurred, at first, only on awaking from sleep, afterwards more frequently; sometimes without any perceptible cause, at other times, and more frequently, when she was vexed or was about to cry. On examining the child accurately, I could discover no deviation from a state of perfect health; the pulse and respiration were natural, skin cool and soft, tongue clean, the breath pure, the appetite good, the bowels regular; the alvine excretions exhibited the appearances usual in healthy children on the breast. She was playful and lively, but was startled unusually by any sudden noise; the four incisor teeth had appeared; the gums were neither swollen nor tender." 603.

The treatment consisted in half a grain of sulph. quininæ every six hours, with free exposure to the cool air. By these means the attacks became less frequent, and, after some days, ceased altogether. She continued in good health.

*Case* 2. Two other cases have fallen under our author's observation, in which the disease was confined to an occasional spasmodic affection of the glottis—no other perceptible derangement of function coexisting with it, nor any dental irritation. In these cases, also, the disease subsided under a mild tonic plan.

3. "A patient whom I had been attending, happened to mention to me incidentally, that her child, about fourteen months old, was in the habit of awaking suddenly from sleep as if alarmed, breathed with difficulty, and made a loud noise like that in a fit of the hooping-cough; she said also, that this occurred occasionally during the day, when the child was cross; she spoke of this lightly, as of an unimportant thing. I requested to see the child; it looked pale and unhealthy; the integuments were soft and flabby; the tongue coated; the bowels relaxed or confined; the stools green and curdy; the child was irritable and nervous, and seemed to suffer from dentition; the extremities were slightly swollen, and the thumbs placed firmly across the palms of the hands. I stated that I thought the child very ill, and that probably convulsions would take place.

Next day I was summoned in haste, and found the child just recovered from a severe paroxysm of general convulsions.

The gums over the projecting teeth were divided; leeches were applied to the temples, cold lotions to the head, and warm fomentations to the extremities; aperient medicines were given; the nurse, whose milk obviously disagreed with the child, was changed; and it was removed from the city to a healthy situation in the country; after one other attack of convulsions, recovery was speedy and complete.

In this case the disease was complicated with derangement of the digestive functions, and also with painful dentition; the convulsive paroxysms, at first partial, increased in severity, and at length became general. The remedies which appeared to be ultimately and permanently efficacious, were, change of air and change of nurse." 605.

4. A fine child, twelve months old, had been subject, for four months, to frequent attacks of what was called "croupy breathing," occurring at first, only in the night, and that rarely—afterwards occasionally in the day. Medical advice was called in, and the treatment consisted of bleeding, emetics, mercurial purgatives, confinement to a heated apartment, flannels, &c.

"When I first saw the child, the struggle in breathing was difficult and protracted, and the face, during the paroxysm, became quite livid: these attacks, which usually terminated with a loud and sonorous inspiration, were excited by very slight causes, such as sudden noises, the removal of the child from the nurse's arms, or any source of irritation or annoyance; and now, instead of going off as formerly, they were, once or twice in the twenty-four hours, terminated by severe and general convulsions, which had all the characters and appearance of an epileptic paroxysm: after one of those, strabismus remained;* there was also a swollen and puffy state of the extremities, with a spastic rigidity of the fingers and toes; the digestive functions were greatly deranged, and the child looked exceedingly ill: it had been weaned for nearly three weeks; a healthy wet-nurse was procured; the gums, rather as a measure of precaution than from necessity, were divided; the temperature of the apartment was lowered, and gradual habituation to the open air adopted; the quantity of clothing with which the child was oppressed, was by degrees diminished, and after a little time, the body was sponged daily with vinegar and water: the child had already been so frequently and copiously bled, that further bleeding was not deemed advisable. Its health improved rapidly, and, restricted in nutriment to the nurse's milk, the bowels soon returned to their natural state; the general convulsions ceased altogether, and the attacks of spasmodic dyspnœa became daily less frequent and severe. As a proof of the completeness of recovery, it may be mentioned, that a few weeks having elapsed, this child was extensively scalded, and yet no convulsive or spasmodic symptom ensued. The child, moderately covered, almost lived in the open air, and, during the whole process of dentition, a succession of fresh wet-nurses was provided. He is now, after the lapse of some years, a fine and healthy boy. In this case, the change of nurse, and the substitution of the tonic for the heating regimen, were attended with results at once speedy and satisfactory." 607.

4. A weak and delicate child, two years of age, had been subject, for

---

* "On the recovery of the child, this symptom gradually disappeared."

some time, to occasional and sudden attacks of dyspnœa, during which the inspirations were short and frequent—the head thrown back—and a sense of suffocation urgent. These attacks, which were frequent and severe, ended in a loud whoop or crow. Between these there were sometimes intervals of several days, when the child appeared tolerably well, but irritable. The paroxysms became more frequent, and excited apprehension.

"On my first visit the child was confined to bed, and laboured under well-marked symptoms of remitting fever; the pulse and respiration were accelerated, skin hot, tongue coated and florid at the edges, the papillæ red and prominent, thirst, and the desire of cold drink urgent, the fæces thin and fetid; there was continual picking at the nose, and the diurnal exacerbations of fever were well marked; the veins of the head appeared unusually turgid; there were frequent attacks of spasmodic dyspnœa, such as have already been described; there was spastic contraction of the flexor muscles of the fingers and toes; the extremities were swollen, and of a slightly livid colour: this child suffered greatly from dentition, the teeth were unusually large, and were protruded at a very late period: on dividing the projecting and vascular gum of one of the molar teeth, some purulent matter was discharged. This child had had congenital cataracts, at the age of three months both eyes had been operated on; severe ophthalmia ensued: he had been copiously bled, and calomel had been given very largely; the child scarcely survived the treatment, and remained afterwards very feeble, emaciated, and delicate. A few leeches were now directed to be applied to the temples, calomel in minute doses was given every third or fourth hour, and occasional doses of Castor oil: the febrile symptoms gradually subsided, the patient recovered slowly, and for some weeks remained without any return of the spasmodic attacks. After some time the child again began to suffer from difficult and painful dentition, and the spasmodic paroxysms recurred with violence: whilst in the act of dividing the gum, the spasmodic attack came on suddenly, and so urgently, that the face became livid, and there was complete opisthotonos; this spasm lasted so long, that respiration was for a time suspended, and for a few seconds, I felt apprehensive lest the child should have died in the struggle. At length, after a long and sonorous inspiration, the spasm subsided, leaving the child in a weak and exhausted state. These formidable attacks recurred frequently, and were followed soon by general convulsions; it was now determined in consultation, that the child should be bled from the arm. The blood was allowed to flow until a perceptible effect was produced upon the system: for ten days afterwards there was not any return of convulsions or spasms: at the end of this time the paroxysms of dyspnœa, terminating sometimes in general convulsions, became again frequent. There now existed extreme emaciation, languor, complete anorexia; fingers and toes rigid; extremities purple and œdematous; nervousness; tremor; and starting at the slightest noises: under these circumstances an injection, consisting of five grains of tobacco-leaves in six ounces of water, was administered: the specific effects of tobacco, in a marked degree ensued, and it was remarkable, that for a month afterwards no convulsive symptoms reappeared. About this period, a slighter recurrence of these symptoms led to the removal of the child from the city to the country, upon which they ceased immediately, and the child improved rapidly in health and strength: recovery appeared now so complete that the child was brought back to a large, and newly-painted house in the city; when after a few hours the spasmodic attacks recurred with violence: on a second removal to the country, they ceased at once; a similar experiment was a second time tried, and with precisely similar results; and it is a curious fact, that two other children were attacked with a similar spasmodic affection in this same newly-painted house; of these, one died in a convulsion, the other, on being sent to the country, recovered: the child, whose case has just been related, had been for years free from any spasmodic affection, but remains delicate, and suffering severely from scrofulous disease." 611.

5. A child, nine months old, was brought to the "Infirmary for Children," labouring under the spasmodic disease in question, but not being informed of this circumstance, only some common remedies were prescribed for improving the digestive functions. In a few days afterwards the author found it violently and generally affected with convulsions, which succeeded each other rapidly, and, in 24 hours, proved fatal.

"The body having been examined twenty-six hours after death, no morbid appearance was discoverable either in the abdominal or thoracic viscera; the mucous membrane of the larynx, trachea, and bronchi, exhibited a perfectly natural appearance; a very large quantity of fluid was found to have been accumulated in the ventricles of the brain; no morbid change had taken place in the brain; this was the only instance in which the disease, under my observation, had terminated in hydrocephalus." 612.

6. The following case was communicated to our author by Dr. Johnson, Professor of Midwifery to the Royal College of Surgeons in Ireland.

Dr. J. was consulted in the case of the child of the Hon. Mrs. P. who was attacked on the third day after birth, with "laryngeal spasm and crowing inspiration," which excited great apprehensions. These attacks returned at intervals, when the child was irritated, and on waking from sleep, until the third month, when they disappeared without any apparent cause. Between the 5th and 6th month, they returned, accompanied by the swellings of the feet and hands described by Dr. Kellie of Leith, and terminated in general convulsions. At this time he cut two incisors in the lower jaw. The attacks of crowing inspiration returned frequently during dentition—sometimes alone, sometimes accompanied by the rigidity of the thumbs and toes, but never with convulsions, until he was cutting the first molar teeth. On examination of the gums, the canine teeth were found making their appearance. The gums were divided—an issue inserted in the nape of the neck, and aperient medicines exhibited. But the attacks were neither diminished in frequency nor violence, until the child was removed to the country, after which he had no return of the disease.

7. The following interesting case was communicated by Mr. Newton, "upon whose soundness and accuracy of observation the fullest reliance may be placed."

"A child, aged nineteen months, of a violent temper, had always been very healthy until it was about seventeen months old, when he had a mild attack of hooping cough, since which he was observed to have occasional fits of difficulty of breathing on awaking from sleep, during which his face became livid; these lasted for some time and were terminated by a long, deep-drawn inspiration, with a crowing noise; this did not excite any alarm in his friends.

On the morning of the day on which he died, he took a hearty breakfast of stirabout, and in about an hour afterwards, he vomited; he was in good spirits and apparent good health until 5 P.M., when he was put into a passion; his breathing suddenly became difficult, his face livid, and he expired in about five minutes.

*Dissection seventeen hours after death.* Extremities stiff, body fat, some lividity about scrotum and posterior parts of the body.

On removing the skull-cap, a large quantity of dark-coloured fluid blood escaped; there was great turgescence of the vessels of the membranes of the brain. The brain was healthy; on removing it, a considerable quantity of blood escaped from the spinal canal.

Mouth and throat.—Uvula, somewhat elongated, tonsils slightly enlarged. The rima glottidis was very much contracted. In the larynx, upper portion of the trachea, and in the œsophagus, numerous small portions of undigested oatmeal were observed; the mucous membrane of the air tubes presented every where a natural appearance. The lungs and right side of the heart were engorged with dark-coloured blood. The mucous membrane of the stomach, about the pylorus, was softened and eroded. The muciparous glands of the small intestines were enlarged. No other disease was observed in any part of the body. On examining the larynx on the following day, the rima glottidis was found to have recovered its natural dimensions." 616.

It is evident that, in this case, the spasmodic closure of the rima glottidis was the immediate cause of death. The turgid state of the cerebral vessels and of the lungs was probably the result of the suffocative struggle. Our author observes that "the symptoms at the commencement are thus proved to be purely spasmodic, and it is only when the disease increases in severity, and when general convulsions arise, that the brain, or its membranes become the seat of the disease." In all the cases which our author has observed of this complaint, the children were of the scrofulous diathesis, or sprung from scrofulous parents. "This," he says, "bears practically on the subject, as it enhances the value of pure air, healthy nutriment, and tonic remedies, in the treatment."

"If we take a survey of the several cases of this disease, which have been stated, we learn that it varies much in degree, and that its complications are numerous. In its mildest and least complicated form, the spasmodic action is confined to the muscles of the glottis, and the treatment consists in improving the general health, and in giving tone to the nervous system. The symptoms in such cases will rarely fail to yield to some of the vegetable or mineral tonics, pure and bracing air, and a well regulated diet: in some cases, I have perceived, I think, advantage to arise from some of the antispasmodic medicines, and amongst these, none has appeared to me more beneficial than the old fashioned medicine, the Tinctura Fuliginis; but when the disease is complicated with painful dentition, derangement of the bowels, or any febrile movement in the system, the primary object of the treatment must be, to remove these accompanying ailments; until this be effected the treatment applicable to the spasmodic affection, though it may mitigate its severity, will fail to eradicate the disease. When the spasmodic symptoms extend themselves, and implicate the muscles of the extremities, the disease assumes a more formidable aspect, and soon, if not checked in its progress, paroxysms of general convulsions will establish themselves: in this stage the membranes of the brain become so frequently engaged, that the utmost vigilance on the part of the practitioner is required to prevent the occurrence of such mischief; yet it must not be lost sight of that the disease, even in its mildest form, is attended with danger: one case is recorded of sudden death during the spasmodic closure of the glottis; this occurred in a child who was otherwise in perfect health, and I have heard of several instances of the same kind. In every stage of the disease, therefore, we should be aware of and guard against the liability to sudden death; all needless sources of irritation should be avoided, and the child closely watched, and carefully held and supported during the paroxysm of dyspnœa. In irritable and passionate children the danger is increased. Dr. Johnson has stated to me, that he has seen a child, in a state of asphyxia caused by this disease, recovered from apparent death by the instantaneous application of artificial respiration." 619.

We must now endeavour to make room for a case, or rather two cases, lately published by Dr. Hall, in the Medical and Physical Journal.

8 and 9. "These cases of the 'peculiar species of convulsions,' described by the late Dr. John Clarke, are quoted and sketched, rather than described, in this paper, chiefly for the sake of the opportunity thus afforded me of presenting a few cursory remarks on this interesting morbid affection of infants. The two little patients were twin brothers, aged nearly nine months. They became affected, nearly simultaneously, by restlessness during the night, and with a hooping or crowing noise in the breathing, three weeks before any symptom occurred which gave alarm.

On the 27th of February, 1824, Master F. L. G. was observed to be indisposed, it was supposed, from cold. The medical friend of the family was sent for in the evening: meantime, however, the infant had fallen asleep, and seemed composed. On the next day, at eleven a.m., the little patient was again visited: it was perfectly lively. All on a sudden it gave a slight hoop. The gums were promptly lanced; three grains of the hydrargyri submurias were prescribed to be administered immediately, and to be repeated in four hours. In the evening it was found that the bowels had been moved freely three or four times, and that no hooping or crowing noise had taken place during the day. It was reported, indeed, that the little patient was quite well. On examining the hand, however, it was found that the thumb was firmly drawn to the palm. In a short time, too, the crowing returned, and it gradually increased. The gums were again freely lanced, and leeches were applied to the throat, which bled sufficiently to induce a little faintness; the hydrargyri submurias was prescribed in the dose of two grains every two hours, and enemata were administered. About two o'clock in the morning, the little patient was attacked by a violent fit of convulsion. He was put into a warm bath immediately, and cold water was dashed into the face until he was restored. I saw this little boy at four o'clock, a. m. There was no return of convulsion until the 2d of March. At eleven o'clock on that day a fit took place, which much exceeded the former one in duration and violence. No return of fit took place until the 5th of March, on which day he had the last. At this time two incisores appeared in the under jaw. The little patient seemed to be better; but the bowels were still torpid.

Each fit was preceded by the crowing noise; but this frequently existed without being followed by a fit. The crowing was attended by a spasmodic action of the muscles situated at the upper part of the throat, and by a difficulty of inspiration. Throughout there were a clenched state of the hands and a contraction of the feet and legs. Sometimes there was difficulty in swallowing, at others not. The bladder was not freely evacuated, except by the aid of emollient clysters.

For a few days before March the 18th this little boy appeared somewhat better. The bowels had acted, and the motions were tinged with bile. The contraction of the hands and feet was relieved. On the 19th, he became restless and tossed his head from side to side. He was relieved by a free evacuation of the bowels. On the 20th, this little boy was obviously very uncomfortable, and there was again a difficulty in swallowing. The 21st was passed comfortably, and he appeared better. The legs were observed to be a little swollen. He passed a good night, was cheerful when he awoke on the morning of the 22d, but died suddenly two hours afterwards, whilst the nurse was giving him a little tea.

On examination post mortem, the general surface of the body was found pale. In the upper jaw there were four teeth (incisores), which pierced the alveolar processes, but were still covered by the periosteum and gums; or at least a probe pushed along the teeth under the membrane was arrested at their edge, though this might be by cicatrix, as the teeth had been many times very completely lanced. There were no teeth appearing on removing the soft parts of the under jaw, except the two which had been cut during life. The scalpel, in all other parts of the jaw, passed down to the bone On raising the dura mater, the tunica arachnoidea was seen extremely distended by subjacent transparent fluid, and interspersed with large arteries and veins, which were very distinguishable by the colour of the contained blood. The tunica arachnoidea was perfectly transparent; on cutting through it, and applying a sponge to remove the subjacent fluid, four drachms by weight were collected. On cutting into the substance of the brain, more red points appeared than usual, and altogether perhaps more fluid exuded. The ventricles contained much serum; at the least an ounce and a half. There were no other morbid appearances in the encephalon; no tumour either in the dura mater or substance of the brain; no abscess. On tracing the spinal marrow three or four inches down, no fluid or other morbid appearance was observed. On making an incision into the thorax, all the viscera were found perfectly healthy, except the pericardium, which contained much more fluid than natural, at least two drachms by weight. The heart was completely empty. The lungs and cavities of the pleura were free from morbid appearances. There was certainly more redness than natural, and that from enlarged vessels, of the pharynx, epiglottis, and rima glottidis; very marked when compared with the adjacent parts; none, however, of the trachea and œsophagus. The viscera and cavity of the abdomen were perfectly healthy, except that the latter contained a very small portion of effused serum."

The other twin was more fortunate, and escaped, though under similar treatment. Dr. H. avers that the disease, in both instances, was precisely similar. We shall introduce the Doctor's remarks in his own words.

"*Remarks.*—I now proceed to make a few reflections upon this singular simultaneous concurrence of fits in twin brothers. I would, in the first place, observe, that the cause of this affection must have been one that was common to the two infants. It might be, 1st, the diet; 2d, the local situation; or 3d, teething. The first causes had, however, obtained, without change, for months previously to the attack; it could scarcely, therefore, be any one of these which should operate so decidedly upon two infants at the same time in so peculiar a manner. The common cause, the operation of which began at the period of the attack, was teething. To this cause, then, the attack was chiefly referred in the first instance. The conjecture was subsequently confirmed by the prompt appearance of the two incisores in the lower jaw of each infant, and by appearances of dentition on the post-mortem examination of the one in whom the disease proved fatal.

I think it important to bear in mind that dentition may be a source of irritation, long before there is any tumour of the gum perceptible to the finger. When the gum begins to be irritated, and stretched by the advancing teeth, the injury may be propagated along the nerve to the brain.

It is also important to remark, that, even after full relief given to the gum by lancing, the injury may continue. Tetanus from a wound does not necessarily cease even after amputation: we have the effect to treat.

It is important to observe, in the next place, that one single fit induces a state of the brain which disposes to a recurrence of the fit. The nervous system does not at once recover from its state of irritation; it remains more susceptible than before.

It is, further, the effect of every kind of fit to induce a gorged state of the blood-vessels of the brain, similar to that observed in the countenance; this condition augments the susceptibility of the brain to further attacks of fits, and may lead to effusion. Even fits of hooping-cough have this effect, and thus frequently lead to fits of other kinds, and to hydrocephalus. I have more than once known a fit of hooping-cough become a fit of convulsion.

Convulsions are, indeed, a multiform affection. The usual form is one which attacks the muscles of ordinary voluntary motion. The species of convulsion described by Dr. J. Clarke, is only remarkable from involving a part of the respiratory system of muscles, especially those about the larynx, and those of inspiration in general. Its best designation would be the *croup-like convulsion*. Another form is that described by the late Dr. Kellie, of Leith, as affecting the hands and feet. I have seen convulsive motions almost confined to the eye, or to some part of the face; but I never thought it worth while to give them a distinct name or epithet.

It is also necessary to keep in view, that there are other sources of convulsion besides irritation. Teething, and a deranged condition of the alimentary canal, are certainly by far the most usual causes of convulsion." 528.

We have seen several cases of this complaint, but never one that proved fatal. The following was put on record, fourteen years ago, in the third volume of the Medico-Chirurgical Journal and Review (monthly series) page 448. It elucidates the treatment which we have always pursued, and hitherto with success.

10. "The patient was a delicate female child, nineteen months old, and cutting three or four teeth at the time. She had been ill eight days before the Reporter saw her; but according to the mother's account, there was little or no variation in the symptoms all the time. When first seen, the child three or four times in the hour was seized with spasmodic affections of the respiratory muscles, consisting of repeated attempts to fill the chest, during which she threw herself back, as in opisthotonos, and appeared as though she would be suffocated. These fits would last ten or twelve minutes, after which the child was somewhat easier, but always fretful and peevish. The backs of the hands and insteps were swollen and hard. The thumbs were rigidly contracted, and locked across the palms of the hands. The toes were bent down towards the soles of the feet, and both wrists and ankles were rigidly bent by the flexor muscles, and kept permanently so. The little patient could take no food: she was slightly feverish; the bowels torpid; and the stools clayey, slimy, and offensive. The eyes looked very heavy and inanimate; the child extremely irritable and restless both by day and by night.

For reasons unnecessary to mention, no very active means were employed for some days, during which the child got worse, and was evidently declining fast. Hydrencephalus or eclampsia being now dreaded, leeches were applied to the temples; calomel and antimonial powder were exhibited thrice a day, and the warm bath was employed in the evening. After the bath and bleeding, the spasms went off for twelve hours entirely, and the child took some food. They returned, however, but not so violently as before, and the convulsive respiration was much relieved. The calomel and antimony were continued, as was the warm bath; and the gums were deeply scarified, wherever a tooth appeared to be coming. From this time the child gradually recovered, and on the 18th day the spasms had entirely disappeared."

Considering that the head is the organ which, if not the original seat of the disease, is the one likely to suffer in the sequel, we should still be inclined to direct our attention to that part, while we scarify the gums, and improve the state of the abdominal secretions. We have considered this curious and interesting subject deserving of a short article in our review, and hope our readers will be of the same opinion.

